# Effect of Repeated Use of Different Types of Scan Bodies on Transfer Accuracy of Implant Position

**DOI:** 10.30476/dentjods.2022.96149.1922

**Published:** 2023-12-01

**Authors:** Ali Mahmoud Hashemi, Mahya Hasanzadeh, Leila Payaminia, Marzieh Alikhasi

**Affiliations:** 1 PhD Student, Dental Implant Research Center, Dentistry Research Institute, School of Dentistry, Tehran University of Medical Sciences, Tehran, Iran; 2 Dental Research Center, Dentistry Research Institute, Dept. of Prosthodontics, School of Dentistry, Tehran University of Medical Sciences, Tehran, Iran; 3 Dental Research Center, Dental Implant Research Center, Dentistry Research Institute, Dept. of Prosthodontics, School of Dentistry, Tehran University of Medical Sciences, Tehran, Iran

**Keywords:** Computer aided design, Computer aided manufacturing, Dental Prosthesis, Implant supported, Impression technique

## Abstract

**Statement of the Problem:**

Some components of implant treatment are reusable. Therefore, possible changes during fixation, removal, and sterilization process should be tested. Many studies have examined the reuse of implant parts, but the impact of repeated use of scan bodies on the accuracy of implant position has not been well investigated.

**Purpose:**

The aim of this *in vitro* study was to compare the effect of repeated use of two different types of scan bodies on the accuracy of implant position.

**Materials and Method:**

In this *in vitro* experimental study, two acrylic resin maxillary models, each with two implant analogues inserted at the site of missing first and second molars were used. Two types of scan bodies including titanium and polyetheretherketone (PEEK) were used for digital impression. Then they were ten times removed and autoclaved for sterilization. The first scan was considered as a reference to be compared with the other next nine scans. Values of linear distance between two scan bodies, diameter changes of each scan body, and three-dimensional linear displacement (ΔR) were measured. These values were compared between the two types of scan bodies using *t*-test (α=.05).

**Results:**

There was significant difference between titanium and PEEK scan bodies regarding inter-implant distance variation (*p*=.006) and diameter change (*p*< .001) in repeated use. However, for the ΔR, there was no significant difference between them (*p*= 0.759).

**Conclusion:**

The results demonstrated that type of scan body could affect the accuracy of implant position transfer after repeated use. PEEK scan body performed better after 9 cycles of reuse in comparison with titanium scan body.

## Introduction

The prerequisite for long term success of osseointegration is to provide passively fitting restorations [ 1 - 2 ]. Many controversies exists about the clinically accepted misfit level, but to keep away from long term complications, 150µm limit was recommended [ 3 ]. Accuracy of transferring implant position from the mouth to laboratory is widely discussed and considered as a determinant factor for final passive fit of restoration [ 4 ]. With the advancement of technology, the computer-aided design (CAD) and computer-aided manufacturing (CAM) process and digital impression have been introduced. Digital impressions improved efficiency as it offered advantages of reduced risk of deformation during transfer and the laboratory phases. Furthermore, by digital technology patient comfort and acceptance increased [ 5 ]. However, in conventional impressions, transfer problems may occur as a result of shrinkage, detachment of the impression material from the tray, different layer thickness, and the impression deforming [ 6 ].

The latest technology in this field is the intraoral scanners used for digital workflow. These scanners are easier to use than conventional techniques and are the first choice of many dentists [ 7 ]. With digital impression technique, there should be no need for commonly used impression copings, but a need for scan bodies, which are used to transfer 3D information about the position and direction of the implants to the virtual cast [ 8 ]. Scan bodies are reliably used for digital impressions [ 9 - 10 ]. They are either monolithic components or a combination of different materials, as aluminum alloy, titanium alloy, polyetheretherketone (PEEK), and various resins [ 11 - 12 ]. Despite the great variability of design and forms of scan bodies, they all consist of three distinct components including scan region, body, and base that form the most apical portion. The scan region is the part, which is scanned, the body extends from the scan region to the base, and the base is the part, which is seated into the connection. The scan region and body usually are made of same material [ 13 - 14 ]. Characteristics of scan bodies including connection type, design, dimension, material, reusability, and compatibility between the surface of scan body and software influence the accuracy of position transferring [ 15 ].

Cost is an important item in choosing a product for health management [ 16 ]. Due to financial issues, many clinicians had to reuse medical equipment [ 17 - 18 ]. In dentistry, there are studies that have concentrated on the reuse of procedural components in orthodontic, endodontic, surgical, and implant treatments [ 17 , 19 - 25 ]. Since implant treatment is relatively expensive, some components are reused. Therefore, possible changes during fixation, removal, and sterilization process should be tested. Their effective performance should also be evaluated [ 26 ]. Many studies have examined the reuse of implant parts [ 27 ], but the impact of repeated use of scan bodies on the veracity of implant position, has not been well investigated. Two research groups had evaluated the reuse of impression copings and scan bodies [ 28 - 29 ]. They recommended the reuse of these parts after cleaning and sterilization. Since PEEK is capable of deformation due to reuse and sterilization process [ 13 ], the purpose of this *in vitro* study is to evaluate the effect of repeated use of two different types of scan bodies on the accuracy of implant position. The null hypothesis was that the accuracy of titanium and PEEK scan bodies are equal after repeated use.

## Materials and Method

**Figure 1:**
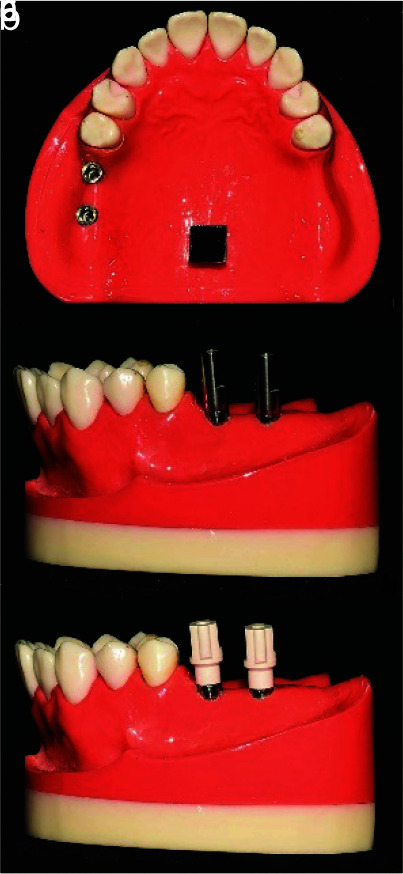
**a:** Maxillary model with two internal connection implant analogues; **b:** Titanium implant scan bodies attached to the implant analogues; **c:** Polyetheretherketone (PEEK) implant scan bodies attached to the implant analogues

In this *in vitro* study, two acrylic resin maxillary models, each with two implant analogues (4.3mm diameter× 11mm length) (Replace Select, Nobel Biocare, Zurich, Switzerland) inserted at the site of missing first and second molars were used. Analogues were fixed into its corresponding holes using auto polymerizing acrylic resin (Technovit 4000, Heraeus, Hanau, Germany). To ensure that the resin polymerization process is completed, the process was stopped for a week. Then, two types of two-pieces scan bodies, both compatible with the implant system, were used. One was titanium based (Doowom, Arum, Daejeon, Korea) and the other was PEEK based (Nt-trading, Scan body 3D-Guide, Karlsruhe, Germany). The connection was in titanium for both (Figure 1). They were attached to implant analogues and torqued to 10N.cm. The models were scanned with intra oral scanner (Trios, 3shape, Copenhagen, Denmark). Then the scan bodies were removed and autoclaved for sterilization. The cleaning project was carried based on the manufacturer’s instructions, which included scrubbing the interior and exterior sides of the scan bodies using a soft-bristled nylon brush for 2 minutes. The copings were then dried on absorbent paper. After that, they were packed and sterilized, using a steam autoclave (Steam Sterilizer A35-B, WEBECO Gmbh & Co, Selmsdorf, Germany) according to DIN standard 13060 and the standard protocol, suggested for the sterilization of surgical and dental equipment [ 30 ]. The process consisted of sterilizing at 134˚C for 10 minutes and then drying for 15 minutes. This process was repeated 9 times for each type of the scan bodies. All scans were performed by an experienced operator, after enough trial scans to find the best scan strategy of the model. The first scan of each type was considered as a reference to be compared with the other next nine scans. For measuring the accuracy of the implant position, the trueness of the scan bodies was evaluated by comparing each scan with the reference one. All scans were saved as standard tessellation language (STL) files.

**Figure 2:**
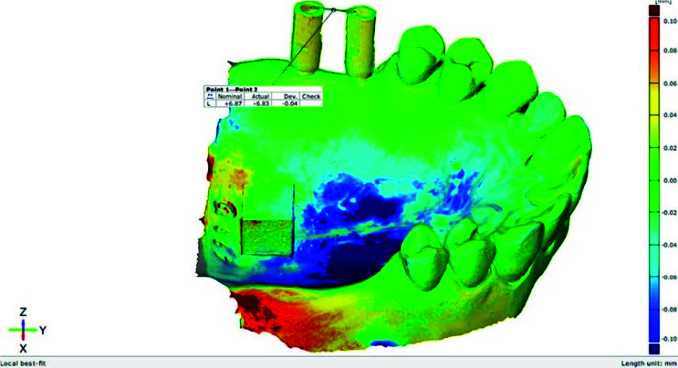
Inter-implant distance measurement

Measurements for all digital datasets were undertaken using GOM software (ATOS Core 80; GOM GmbH, Braunschweig, Germany). Each scan was superimposed to the reference scan (Ref) based on the geometry of remaining teeth with local best-fit option in the software. A CNC milled cube was attached to the model in order to define three-dimensional (3D)-coordinate origin (point 0,0) in all scans. A cylinder and a plane best fitted to the external and occlusal surface of each scan body were defined. The central axis of each cylinder was specified and its intersection with the occlusal plane was marked as point A and B for anterior and posterior implants, respectively. Three-dimensional position of each point (R) was calculated with x, y, and z coordinates $\left(\sqrt{X_{2}+Y_{2}+Z_{2}}\right)$ and ΔR was defined as R–R_Ref._ Inter-implant distance variation was obtained by calculating the linear distance between the two scan bodies from point A to point B (Figure 2). Diameter changes of the cylinder best fitted to the scan body were calculated to indicate the diameter changes of each scan body.

All statistical analyses were performed using SPSS 18.0.0 (SPSS Inc., Chicago, IL). The *p* Values less than 0.05 were considered statistically significant. A sample Size of 10 in each group achieve 75.288% power to reject the null hypothesis. All tests were two-sided. Shapiro-Wilk test was applied to examine the normality assumption. The mean and standard deviation values were reported for dependent variables including inter-implant distance variation, diameter change, and ΔR. T-test was considered to compare the two scan bodies.

## Results

**Figure 3:**
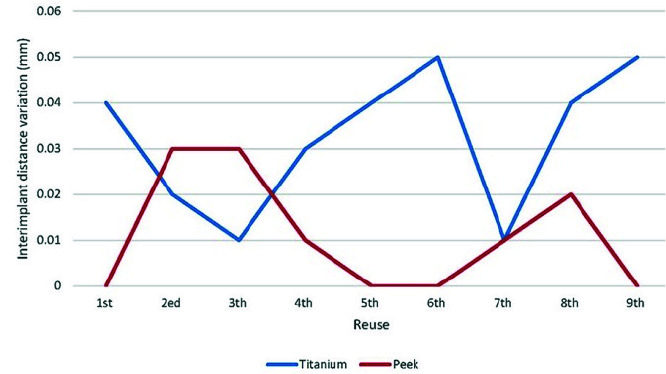
Comparison of inter-implant distance variation between titanium and polyetheretherketone (PEEK) scan bodies during repeated use

**Figure 4:**
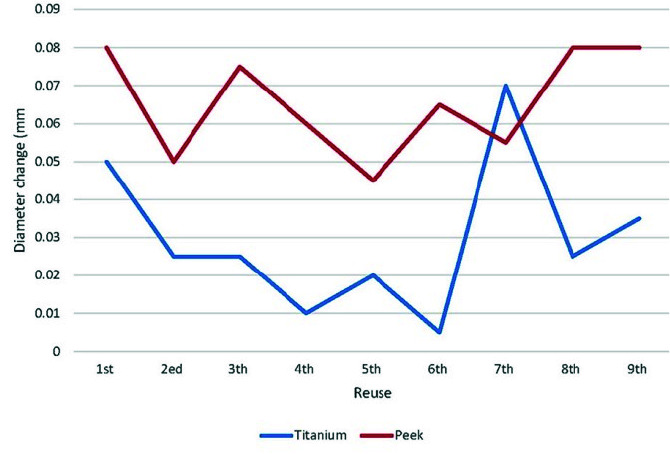
Comparison of diameter change between titanium and polyetheretherketone (PEEK) scan bodies during repeated use

**Figure 5:**
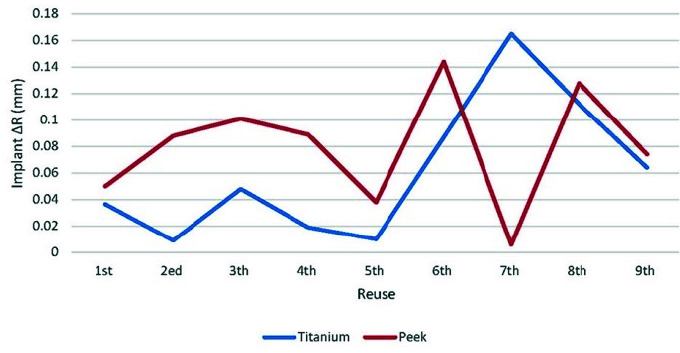
Comparison of ∆R between titanium and polyetheretherketone (PEEK) scan bodies during repeated use

Ten scans were obtained from repeated use of each type of scan body. The mean and standard deviation for inter-implant distance variation, diameter change, and ΔR are presented in Table 1. The results indicated that there was significant difference between titanium and PEEK scan bodies regarding inter-implant distance variation (*p*= .006) and diameter change (*p*< .001) in repeated use of them. The inter-implant distance variations were more in titanium than PEEK scan bodies (mean difference = 0.021mm), while the titanium scan bodies had less diameter changes than PEEK ones after repeated use (mean difference= 0.037mm). However, for the ΔR, there was no significant difference between titanium and PEEK scan bodies (*p*= 0.759). Figures 3 to 5 show the graphical representation of inter-implant distances, changes in diameter of scan bodies, and ΔR with respect to autoclave cycles. 

**Table 1:** Descriptive values for inter-implant distance variations, diameter changes, and ΔR of the groups

Outcomes	Scan body		*p* Value
	Titanium	PEEK	
Inter Implant Distance changes	0.032±0.016	0.011±0.012	*p*= .006
Diameter changes	0.029±0.020	0.066±0.014	*p*< .001
ΔR	0.069±0.052	0.080±0.044	*p*= 0.759

Data are expressed as mean±SD. PEEK: polyetheretherketone

## Discussion

The results of this study rejected the null hypothesis. It was demonstrated that the inter-implant distance variations were more in titanium than PEEK scan bodies. The results further indicated that titanium scan bodies had less diameter changes than PEEK scan bodies after repeated use. However, regarding the three-dimensional linear displacement (ΔR) there was no significant difference between them.

There are studies that have focused on the possibility of reusing some implant parts [ 17 , 25 , 28 , 29 , 31 - 33 ]. The effects of reused cover screws on clinical outcomes were evaluated by Schwartz *et al*. [ 17 ]. They gathered that despite reusing cover screws could result in surface properties alteration, it would not adversely affect clinical outcomes. A systematic review indicated that common method used for cleaning and sterilization of healing abutments and cover screws might not result in the complete removal of contaminants. However, it would not cause any biologic or mechanical complications [ 34 ]. Other studies evaluating reuse of implant components have shown that cleaning, sterilization, and mechanical changes during insertion and removal can alter the surface morphology of implants, which result in variations in differentiation and osteoblastic growth [ 25 , 35 ]. However, it is not likely to be an issue with impression copings, as they have no constant contact with hard and soft tissues [ 25 ]. Browne *et al*. [ 31 ] reported that by sterilizing used impression copings, they did not show any particular deformation and were comparable to new copings. Alikhasi *et al*. [ 28 ] and Babu *et al*. [ 32 ] also indicated that impression copings could be cleaned, sterilized, and reused up to 10 and 12 cycles, respectively, without meaningfully decreasing the impression accuracy. In another study, a more number of reusing cycles was studied. Gallardo *et al*. [ 33 ] evaluated the effect of reusing and changing impression copings on impression accuracy. They concluded that after 30 times of cleaning and sterilization, impression copings that were modified by airborne-particle abrasion and polyvinyl siloxane (PVS) adhesive showed less impression inaccuracy than that unmodified impression copings. However, they were all still clinically acceptable [ 33 ]. 

Sawyers *et al*. [ 29 ] investigated the effect of several using of impression copings and scan bodies on implant cast accuracy. In that study, an implant stone cast with two bone level internal connection implant analogues was used to make ten conventional and ten digital impressions. They reported no significant differences between the impressions by reusing impression copings or scan bodies up to 10 times [ 29 ]. However, their measurement method was different from present study. They used a non-implant related reference point, the right mandibular canine tip, in relation to the linear z-axis measurement. In the present study, superimposing of the scans was based on best fit of the teeth. Moreover, the exact point of intersection between cylinder axis and the upper plane was defined for measurements. Moreover, it is worth mentioning that contrary to the present study, Sawyers *et al*. [ 29 ] simulated indirect scanning using a laboratory scanner. Thus, the scan bodies were detached, removed from the cast, and reattached without being subjected to sterilization. Their results might not be generalized to reuse of scan bodies in direct intraoral scanning. Stimmelmayr *et al*. [ 15 ] evaluated the reproducibility of scan body fit on both stone and polymer models by reusing the scan bodies up to 10 times. They reported a better repositioning ability of the scan body on lab analogues than on original implants. They suggested that companies should reduce machining tolerance to increase the renewable fit of the scan bodies in the original implants. 

The optical properties of the scan region material might potentially affect the number of points detected by a scanner [ 36 ], while the mechanical properties of the base material could influence the fit and wear resistance of the scan body connection, particularly when reusing the scan body [ 37 ]. In present study, the more inter-implant distance variations in titanium scan bodies may be related to their more reflective surface. The more diameter changes in PEEK scan bodies with titanium connection could be due to the surface deterioration during sterilization process and the reason why no significant difference was found in three-dimensional linear displacement (ΔR) between these two scan bodies might be because of their same titanium connection. Arcuri *et al*.[ 33 ] [ 37 ] investigated the effect of implant scan body material on the accuracy of full-arch digital impression. Three intraoral scan bodies including PEEK, titanium, and PEEK with a titanium base were used. They showed that intraoral scan accuracy was influenced by the scan body material. PEEK exhibited the best outcomes on both angular and linear measurements, followed by titanium, and PEEK with a titanium base was the less accurate [ 37 ]. They explained that the worst performance of the PEEK with a titanium base scan body could be attributed to the possible microscopic mismatch between its two components. Their different study method in which scan bodies were not reused could have possibly lead to different results [ 37 ]. 

The effect of sterilization cycles on dimensional stability of PEEK have been studied for medical devices. Kumlar *et al*. [ 38 ] reported 6% decrease in lateral dimension of a clip after 30 cycles of sterilization. However, as dimensional change is highly related to the shape of device. The result of mentioned study could not be extended to scan bodies.

Some factors such as inter-implant distance and diameter changes have an equal effect on single-unit restoration. In the case of using multi-unit restorations, the effects of these factors are dissimilar [ 10 , 12 ]. In our study two implants were inserted; with the change in the diameter (smaller or bigger diameter of scan body), the center of the scan body remains constant and there is no problem in the path of insertion of the two-unit restorations. However, inter-implant distance has a direct effect on the insertion of the two-unit restorations. Therefore, the lower inter-implant distance variation in PEEK is clinically more important.

In this study, a colorful map of deviation of the scans from the reference scan was also presented. The mean diameter changes of titanium and PEEK scan bodies were 0.03 and 0.06 mm, respectively. However, this deviation was consistent during repeated use in both types of scan bodies. All expected changes after repeated use is assumed to be from displacement or deformation of scan body. Because of consistent changes of diameter, it could be assumed that the changes in position are related to displacement rather than deformation of scan bodies. The inter-implant distance variations were higher in titanium compared to PEEK scan bodies. This higher variation in titanium scan bodies might be attributed to undesired reflective properties of the metal. Neither the scan body nor the scanner manufacturer's guideline recommended using opacifiers. However, it seems that dusting the scan region with a light coating of titanium dioxide powder, or sandblasting it with alumina powder would logically reduce surface reflection during scan, which might lead to higher accuracy. Arcuri *et al*. [ 37 ] evaluated the effect of scan body material and reported that PEEK scan bodies have the highest accuracy followed by titanium and titanium-PEEK. The material of matting surface could also influence the fitting of scan body to the implant and be responsible for wear after repeated tightening. In this study both scan bodies have titanium base and differences was related to the material of body and scan region of scan bodies.

The main aim of this study was to investigate the effect of repeated use of two different types of scan bodies on the accuracy of implant position. Accordingly, an intraoral scanner, instead of a more accurate scanner like a desktop scanner or an industrial one, was used to make the first (reference) scan. Based on the literature, digital impression on dental implants provides a comparable accuracy compared with conventional impression technique [ 39 ]. Digital impressions using scan bodies are shown to have similar accuracy as conventional impressions [ 40 - 41 ]. Therefore, digital impression using an intraoral scanner was considered as an acceptable method. Indeed, regardless of how accurate the first scan was, we aimed to assess possible changes of the next cycles compared with the first one. It is significant to corroborate that the outcomes were limited to 10 cycles of reusing the scan bodies and might not be suitable to clinical situations. Accordingly, more research is needed to compare the effect of repeated use of other different types of scan bodies with a more number of reusing cycles on the accuracy of implant position. 

## Conclusion

Due to the limitations of this study, it is concluded that the type of scan body could affect the accuracy of implant position transfer after repeated use. PEEK scan body had a better performance after 10 cycles of reuse in comparison with titanium scan body.
